# Reply: Breast cancer, human papilloma virus and sexual activities

**DOI:** 10.1038/sj.bjc.6604104

**Published:** 2008-01-22

**Authors:** J S Lawson, W K Glenn, N J Whitaker

**Affiliations:** 1 School of Biotechnology and Biomolecular Sciences, University of New South Wales, SYDNEY, New South Wales 2052, Australia

There are now five studies, including the report of Akil and co-workers in this issue of the *Br J Cancer*, for which the relationship between age of women and high-risk human papilloma virus (HPV)-positive breast cancer, has been published. Three of these studies report that the age of women with HPV-positive breast cancer is significantly younger than the women with HPV-negative breast cancer. On an average, Greek women with HPV-positive breast cancer were 15 years, Australian women 8 years, and Canadian and Syrian women were 11 years younger than those with HPV-negative breast cancer (Kroupis *et al*, 2006; Lawson *et al*, 2006; Akil *et al*, 2007). Two studies report no difference in the age of women with HPV-positive or -negative breast cancer (Hennig *et al*, 1999; Damin *et al*, 2004). Some details of these studies are shown in Table 1.

Based on the younger age of some women with HPV-positive breast cancer, and the higher incidence of HPV-positive cervical cancer among younger women with multiple sexual partners, we have hypothesised that high-risk HPVs may be transmitted to the breast during sexual activities (Lawson *et al*, 2006).

The underlying assumption to these observations and hypotheses is that high-risk HPVs may have a causal role in some breast cancers. Therefore, a brief overview of the relevant evidence is of value.

The presence of high-risk HPV DNA in breast tumours has been shown, mainly by PCR analyses, in 11 out of 13 studies conducted in a various countries (reviewed by Lawson *et al*, 2006; plus a recent positive study by Yasmeen *et al*, 2007; a negative study by Lindel *et al*, 2007). Tumours of the breast nipple appear to have histological characteristics similar to HPV-positive cervical cancer (de Villiers *et al*, 2005).

Similar high-risk HPVs have been identified in breast tumours and cervical cancer that have occurred in the same women (Hennig *et al*, 1999; Widschwendter *et al*, 2004).

There is immortalisation and preneoplastic transformation of normal breast epithelial cells by HPVs (Band, 1995).

High-risk HPVs, in particular HPV types 16, 18, 31, 33 and less commonly additional types, are the accepted cause of cervical and other ano-genital cancers (IARC, 1995). Less well known is the likely causal role of high-risk HPVs in cancers of the head and neck (van Houten *et al*, 2001). The biological mechanisms by which HPVs are tropic and oncogenic to epithelial cells is reasonably well known from studies of cervical oncogenesis. These mechanisms include the presence and genomic integration of HPV DNA in epithelial tumours, the expression of the HPV E6 oncogene in the tumour where it binds to and degenerates the tumour suppressor p53 gene allowing unregulated cell proliferation to occur (zur Hausen, 2002). Hormones play a central role on HPV oncogenesis. Estrogens synergise with HPV oncogenes to cause cervical cancer (Brake and Lambert, 2005) and the regulatory region of HPV 16 contains DNA sequences that are responsive to glucocorticoid hormones (Gloss *et al*, 1987).

Because of the presumably low viral load, PCR analyses for HPV on both formalin-fixed and fresh-frozen breast tumour specimens are difficult and very dependent on the details of the methods used. For example, the negative outcomes by Lindel *et al* (2007) have probably been because they used the incorrect PCR primers (Damin *et al*, 2007; Yasmeen *et al*, 2007). In our own studies, the identification of HPV DNA proved to be difficult and required additional amplification of the DNA before PCR and the use of SYBR Green I to optimise detection (Kan *et al*, 2005).

The main means of transmission of HPVs appears to be by surface contact. The human papilloma virions are released when the cornified envelope of cells desquamate (Bryan and Brown, 2001). The possibility of transmission of high-risk HPVs during sexual activity is demonstrated by the high prevalence of these viruses in male and female genital organs. The prevalence of high-risk HPVs in male genital organs varies by population and methods of detection. The prevalence of high-risk HPVs in the penile glans, penile shaft, prepuce and scrotum is between 5 and 50%, the perianal area 0–33%, semen 2–83% and urine up to 7% (Dunne *et al*, 2006). High-risk HPVs are also present in normal, benign hyperplastic and malignant prostate tissues (Zambrano *et al*, 2002). The prevalence of high-risk HPVs in females varies between populations and dramatically so between age groups, with the prevalence in near normal cervical smears from UK women 61% at ages 20–24 decreasing to 14–15% in those over 50 years (Cotton *et al*, 2007).

What meaning might be given to these observations? In our view, the evidence that high-risk HPVs may have an aetiological role in human breast cancer is substantial but far from conclusive. Obviously further work needs to be done.

A working hypothesis is that high-risk HPVs may be involved in the initiation of breast cancer among younger women. **The Editor now considers correspondence on this publication closed.**

## Figures and Tables

**Table 1:** Average age of women with HPV-positive and HPV-negative breast cancer

**Study**	**Population**	**HPV type**	**Age HPV +**	**Age HPV--**	***P*-Value for age difference**
Hennig *et al*, 1999; *n*=107	Norway	16	50.8	49.4	0.659, NS
Damin *et al*, 2004; *n*=101	Brazil	16/18	56.5	55.9	0.806, NS
Kroupis *et al*, 2006; *n*=107	Greece	16	38	55	0.001, S
Lawson *et al*, 2006; *n*=50	Australia	18	55.6	63.8	0.049, S
Akil *et al*, 2007	Canada/Syria	16	46.5	57.5	0.05, S

NS=not significant; S=significant at 95% level.

## References

[bib1] Akil N, Kassab A, Yasmeen A, Darnel AD, Bismar TA, Al Moustafa A-E (2007) Human breast cancer and sexual activities. Br J Cancer (in press) 10.1038/sj.bjc.6604103 PMC2361438 18219293

[bib2] Band V (1995) Preneoplastic transformation of human mammary epithelial cells. Semin Cancer Biology 6: 185–192 10.1006/scbi.1995.0015 7495987

[bib3] Brake T, Lambert PF (2005) Estrogen contributes to the onset, persistence and malignant progression of cervical cancer in a human papilloma virus-transgenic mouse model. Proc Natl Acad Sci USA 102: 2490–2495 15699322 10.1073/pnas.0409883102 PMC548999

[bib4] Bryan JT, Brown DR (2001) Transmission of human papillomavirus type 11 infection by desquamated cornified cells. Virology 281: 35–42 11222093 10.1006/viro.2000.0777

[bib5] Cotton SC, Sharp L, Seth R, Masson LF, Little J, Cruishank ME, Neal K, Waugh N (2007) Lifestyle and socio-demographic factors associated with high risk HPV infection in UK women. Br J Cancer 97: 133–139 17519896 10.1038/sj.bjc.6603822 PMC2359671

[bib6] Damin AP, Damin DC, Alexander CO (2007) HPV infection and breast cancer. Breast 16: 222 17398094 10.1016/j.breast.2007.02.005

[bib7] Damin APS, Karam R, Zettler CG, Caleffi M, Alexandre CO (2004) Evidence for an association of human papillomavirus and breast carcinomas. Breast Cancer Res Treat 84: 131–137 14999143 10.1023/B:BREA.0000018411.89667.0d

[bib8] De Villiers E-M, Sandstrom RE, zur Hausen H, Buck CE (2005) Presence of papillomatous sequences in condylomatous lesions of the mamillae and in invasive carcinoma of the breast. Breast Cancer Res 7: R1–R11 15642157 10.1186/bcr940 PMC1064094

[bib9] Dunne EF, Nielson CM, Stone KM, Markowitz LE, Giuliano AR (2006) Prevalence of HPV infection among men. J Infect Dis 194: 1044–1057 16991079 10.1086/507432

[bib10] Gloss B, Bernard HU, Seedorf K, Klock G (1987) The upstream regulatory region of the human papilloma virus 16 contains an E2 protein independent enhancer which is specific for cervical carcinoma cells and regulate by glucocorticoid hormones. EMBO J 6: 3735–3743 2828035 10.1002/j.1460-2075.1987.tb02708.x PMC553844

[bib11] Hennig EM, Suo Z, Thoresen S, Holm R, Kvinnsland S, Nesland JM (1999) Human papillomavirus 16 in breast cancer of women treated for high grade cervical intraepithelial neoplasia (CIN III). Breast Cancer Res Treat 53: 121–135 10326789 10.1023/a:1006162609420

[bib12] International Agency for Research on Cancer (IARC) (1995) Monographs on the Evaluation of Carcinogenic Risks to Humans: Human Papillomaviruses. Vol 64, Lyon, France

[bib13] Kan C-Y, Iacopetta BJ, Lawson JS, Whitaker N (2005) Identification of human papilloma virus DNA gene sequences in human breast cancer. Br J Cancer 93: 946–948 16222323 10.1038/sj.bjc.6602778 PMC2361649

[bib14] Kroupis C, Markou A, Vourlidis N, Dionyssiou-Asteriou A, Lianidou ES (2006) Presence of high-risk human papillomavirus sequences in breast cancer tissues and association with histopathological characteristics. Clin Biochem 39: 727–731 16780823 10.1016/j.clinbiochem.2006.03.005

[bib15] Lawson JS, Kan C-Y, Iacopetta BJ, Whitaker NJ (2006) Are some breast cancers sexually transmitted? Br J Cancer 95: 1708 17133265 10.1038/sj.bjc.6603502 PMC2360761

[bib16] Lindel K, Forster A, Altermatt HJ, Greiner R, Gruber G (2007) Breast cancer and human papillomavirus (HPV) infection: no evidence of a viral etiology in a group of Swiss women. Breast 16: 172–177 17088061 10.1016/j.breast.2006.09.001

[bib17] van Houten VM, Snijders PJ, van den Brekel MW, Kummer JA, Meijer CJ, van Leeuwen B, Denkers F, Smeele LE, Snow GB, Brakenhoff RH (2001) Biological evidence that human papillomaviruses are etiologically involved in a subgroup of head and neck squamous cell carcinomas. Int J Cancer 93(2): 232–235 11410871 10.1002/ijc.1313

[bib18] Widschwendter A, Brunhuber T, Wiedemair A, Mueller-Holzner E, Marth C (2004) Detection of human papillomavirus DNA in breast cancer of patients with cervical cancer history. J Clin Virol 31: 292–297 15494272 10.1016/j.jcv.2004.06.009

[bib19] Yasmeen A, Ricciardi R, Kassab A, Bismar TA, Al Moustafa A-E (2007) High risk HPVs in human breast cancer and normal mammary tissues. Breast 16: 445 17618117 10.1016/j.breast.2007.05.011

[bib20] Zambrano A, Kalantari M, Simoneau A, Jensen JL, Villarreal LP (2002) Detection of human polyoma viruses and papilloma viruses in prostatic tissue reveals the prostate as a habitat for multiple viral infections. Prostate 53: 263–276 12430138 10.1002/pros.10157

[bib21] zur Hausen H (2002) Papillomaviruses and cancer: from basic studies to clinical application. Nat Rev Cancer 2: 342–350 12044010 10.1038/nrc798

